# Management of Green Mold Disease in White Button Mushroom (*Agaricus bisporus*) and Its Yield Improvement

**DOI:** 10.3390/jof8060554

**Published:** 2022-05-24

**Authors:** Suhail Altaf, Shaheen Kousar Jan, Shafat Ahmad Ahanger, Umer Basu, Roaf Ahmad Rather, Owais Ali Wani, Farahnaz Rasool, Muntazir Mushtaq, Mohamed Taha Yassin, Ashraf Abdel-Fattah Mostafa, Abdallah M. Elgorban, Ehab El-Haroun, Ahmed M. El-Sabrout, Ryan Casini, Hosam O. Elansary

**Affiliations:** 1 Division of Plant Pathology, Sher-e-Kashmir University of Agricultural Sciences and Technology-Kashmir, Shalimar, Srinagar 190025, India; suhailaltaf474@gmail.com (S.A.); shaheenkauser10@gmail.com (S.K.J.); samaanjum786@gmail.com (F.R.); 2 Division of Plant Pathology, FoA, Sher-e-Kashmir University of Agricultural Sciences and Technology of Kashmir, Wadura Sopore 193201, India; shafatahanger99@gmail.com (S.A.A.); ratherrouf99@gmail.com (R.A.R.); 3 Division of Plant Pathology, Sher-e-Kashmir University of Agricultural Sciences and Technology, Jammu 180009, India; 4 Division of Soil Science and Agricultural Chemistry, Faculty of Agriculture, Sher-e-Kashmir University of Agricultural Sciences and Technology of Kashmir, Wadura Sopore 193201, India; owaisaliwani@gmail.com; 5 ICAR-National Bureau of Plant Genetic Resources, Division of Germplasm Evaluation, New Delhi 110012, India; 6 Botany and Microbiology Department, College of Science, King Saud University, Riyadh 11451, Saudi Arabia; myassin2.c@ksu.edu.sa (M.T.Y.); asali@ksu.edu.sa (A.A.-F.M.); aelgorban@ksu.edu.sa (A.M.E.); 7 Animal Production Department, Faculty of Agriculture, Cairo University, Cairo 12613, Egypt; elharoun@gmail.com; 8 Department of Applied Entomology and Zoology, Faculty of Agriculture (EL-Shatby), Alexandria University, Alexandria 21545, Egypt; elsabroutahmed@alexu.edu.eg; 9 School of Public Health, University of California, Berkeley, 2121 Berkeley Way, Berkeley, CA 94704, USA; ryan.casini@berkeley.edu; 10 Plant Production Department, College of Food and Agriculture Sciences, King Saud University, Riyadh 11451, Saudi Arabia

**Keywords:** *Agaricus bisporus*, compost, disease control, fungitoxicants, *Trichoderma harzianum*

## Abstract

Mycoparasites cause serious losses in profitable mushroom farms worldwide. The negative impact of green mold (*Trichoderma harzianum*) reduces cropping surface and damages basidiomes, limiting production and harvest quality. The goal of the current study was to evaluate new generation fungicides, to devise suitable management strategies against the green mold disease under prevailing agro-climatic conditions. Six non-systemic and five systemic fungitoxicants were evaluated for their efficacy against pathogen, *T. harzianum*, and host, *Agaricus bisporus*, under in vitro conditions. Among non-systemic fungicides, chlorothalonil and prochloraz manganese with mean mycelium inhibition of 76.87 and 93.40 percent, respectively, were highly inhibitory against the pathogen. The least inhibition percentage of 7.16 of *A. bisporus* was exhibited by chlorothalonil. Under in vivo conditions, use of captan 50 WP resulted in a maximum yield of button mushroom of 14.96 kg/qt. So far, systemic fungicides were concerned, carbendazim proved extremely inhibitory to the pathogen (89.22%), with least inhibitory effect on host mycelium (1.56%). However, application of non-systemic fungitoxicants further revealed that fungicide prochloraz manganese 50 WP at 0.1–0.2 percent or chlorothalonil 50 WP at 0.2 percent, exhibited maximum disease control of 89.06–96.30 percent. Moreover, the results of systemic fungitoxicants showed that carbendazim 50 WP or thiophanate methyl 70 WP at 0.1 percent reduced disease to 2.29–3.69 percent, hence exhibiting the disease control of 80.11–87.66 percent. Under in vivo conditions, fungicide myclobutanil at 0.1 percent concentration produced the maximum button mushroom production of 12.87 kg/q.

## 1. Introduction

Mushroom cultivation is one of the great microbial methods of profitable importance for vast scale utilizing of agro-waste under regulated conditions, and hence its cultivation relieves the pressure on arable land. Mushrooms are saprobic macrofungi that continue to grow and develop on decomposed organic materials. These macrofungi have attracted the curiosity of scientists and the public due to their vital role in forest ecology, food and pharmaceutical industries, bio-degradation and having marvelous health advantages [1]. They make a fantastic complement to our diet since they are high in critical vitamins and minerals, and the Romans referred to them as “Food of the Gods”. These fungi have hypocholesterolemic, hypoglycemic, hypotensive, anticancer (carcinostatic), hypolipidemic, immune modulatory, haematoprotective, antiviral, antibacterial and antifungal properties, including blood sugar and blood pressure reduction attributes properties. Mushroom and truffle total output have been on the rise in recent years. In the previous 20 years, annual output volume has increased by an average of 5.5 percent, nearly doubling to close to 11.9 million MT in 2019 [2]. However, numerous factors, such as the ratio of carbon and nitrogen content, temperature, luminosity, water activity, air composition, humidity, pH as well as the casing material and quality of growing substrate/compost play key roles in mushroom cultivation, having substantial influence on the formation and development of fruiting bodies [3,4]. In addition, a variety of bacterial, fungal and viral pathogens infest the crop, resulting in either limited or complete collapse of the crop or a minimum degradation of quality of the produce. But among the major fungal diseases including wet bubble, cobweb, dry bubble and competitor molds, green mold disease caused by *Trichoderma* spp. [5] has been noted to cause considerable damage, with production losses around 63–65 percent in mushroom farming [6]. Despite the large number of *Trichoderma* species (T. crassum Bissett, *T. koningii* Oudem., *T. citrinoviride* Bissett, *T. spirale* Bissett, *T. longibrachiatum* Rifai, *T. hamatum* (Bonord.) Bainier) found in mushroom compost [6], green mold outbreaks in the cultivation of *A. bisporus* had been attributed initially to *T. harzianum* [4,7]. During the last few years, the recurrent epiphytotics of the disease were observed under temperate agroclimatic conditions of the Kashmir region, as its existence adopts greater importance causing an enormous mushroom crop losses and has necessitated us to generate information on some important aspects of the disease. Taking into consideration the devastating nature of disease in growing mushroom crops, successful management of the disease warrants monitoring, since every single method lacks the flexibility and needed to destroy the diverse population of pathogens over an extended period of time for the harmonious use of various chemicals. Although several aspects of the disease have been studied elsewhere in the world, yet meager information about its occurrence and management is available in India, particularly in Kashmir region, where agro-climatic conditions are congenial for mushroom cultivation and are absolutely distinct from rest of the country. An attempt was therefore made to evaluate new generation fungicides for suitable management strategies under prevailing agro-climatic conditions of the Kashmir region.

## 2. Materials and Methods

### 2.1. Isolation, Identification and Pathogenicity of Green Mold Pathogen

During the present study, green mold pathogen (*T. harzianum*) was isolated from infected casing and infected sporophores, displaying typical symptoms, by routine pathological techniques of Holliday, 1980 [8], collected during a survey of mushroom production units located at Baramulla, Pulwama and Budgam of Kashmir valley. The isolated *T. harzianum* was tested for its aggressiveness towards the mushroom *A. bisporus* under in vivo conditions. To prove the pathogenic nature of isolated *T. harizianum* as a green mold causing agent, two sets of experiments were carried out in polybags of 5 kg compost capacity. The polybags were disinfected with 2 percent formalin followed by washing thrice with sterilized distilled water (SDW). The bags were filled with pasteurized compost, spawned and then kept in spawn run room at 24 ± 2 °C. In the first experiment, conidial suspension (1 × 10^4^/mL SDW) of isolated *T. harizianum*, inoculated in sterilized casing mixture at the time of casing. In the second experiment, isolated *T. harizianum* was inoculated on healthy pinheads and fruit-bodies via conidial suspension (1 × 10^4^/mL SDW) and the mycelial discs from the active culture plate to observe mold symptom development. After inoculation, the bags were kept in an isolated room at a temperature of 21 ± 1 °C with relative humidity of 85 percent. Polybags without pathogen inoculation, maintained under similar conditions, served as a control and were kept apart to avoid contamination. Both the experiments of pathogenicity tests were closely monitored for symptom development.

The morphological cultural characteristics, *viz*., mycelial, colour and growth; shape, size, color and septation of hyphae and conidiophores, conidia and phialides of the isolated *T. harizianum* on host *A. bisporus* and in artificial culture were examined. Morphological and cultural characteristics of the isolated *T. harizianum* were compared with standard descriptions of *Trichoderma* sps given by Rifai (1969) [9]. To further support the identification, *T. harizianum* was also reconfirmed from ITCC, IARI, New Delhi with accession No. 11148.19.

### 2.2. In Vitro Fungitoxicant Evaluation

The effect of six commercial non-systemic fungitoxicants, namely chlorothalonil, mancozeb, captan, prochloraz manganese, zineb and propineb, at 50, 75, 100, 200 and 500 μg mL^−1^ concentrations and five systemic fungitoxicants, namely myclobutanil, carbendazim, hexaconazole, thiophanate methyl and difenoconazole at concentrations of 10, 25, 50, 75 and 100 μg mL^−1^ were evaluated against the mycelial growth of green mold pathogen and host by using the poisoned food technique [10] (Table 1).

Using the poisoned food technique [10], six non-systemic and five systemic fungitoxicants were tested in vitro against mycelial growth of green mold pathogen (*T. harzianum*) and host (*A. bisporus*). PDA media was amended with the fungitoxicants by dissolving 25 mL of double strength test fungitoxicant concentration in 25 mL of sterilized PDA in 150 mL Erlenmeyer flask. Stock fungitoxicant solutions were made in sterilized distilled water by dissolving double the amount of fungitoxicants necessary in a 100 mL volume of sterilized distilled water. The calculated amount of prepared stock solution was poured to double concentrate sterilized potato dextrose agar media to achieve final non-systemic fungitoxicant concentrations of 50, 75, 100, 200 and 500 μg mL^−1^. A similar approach was used for systemic fungitoxicants to achieve the concentrations of 10, 25, 50, 75 and 100 μg mL^−1^. The amended PDA media was aseptically poured into sterilized Petri-plates. *T. harzianum* (5 old culture) mycelial discs (5 mm) were inoculated aseptically to the center of petri-plates. *T. harzianum* inoculated on PDA media containing no fungitoxicants, served as control. A similar method was adopted for evaluation of fungitoxicants against the mycelial growth of host *A. bisporus* (14 day old culture). The inoculated were incubated at 25 ± 2 °C for seven consecutive days. Each treatment was replicated thrice. Data on mycelial growth development of three replications were recorded after seven days of incubation. Mycelial growth inhibition as index of fungicidal efficiency was calculated for each tested fungitoxicants [4].

(1) $$P\;I=\frac{C-T}{C}\times 100$$

where *PI* = Percent inhibition; *T* = Radial growth of fungus (mm) observed in the presence of the tested fungicide; *C* = Radial growth of fungus in control (mm)

### 2.3. In Vivo Evaluation of Fungitoxicants

From the in vitro evaluation, fungicides that showed best results and gave minimum inhibition to *A. bisporus* mycelium were assessed in vivo. Systemic fungitoxicants, *viz*., carbendazim 50 WP, myclobutanil 10 WP and difenoconazole 25 EC at 0.025, 0.05 and 0.1 and non-systemic fungitoxicants, viz., prochloraz manganese 50 WP, chlorothalonil 75 WP and captan 50 WP at 0.05, 0.1 and 0.2 percent concentrations, were evaluated against the green mold disease of *A. bisporus*. A long method of composting [11] was adopted and ingredients as per Formula SKI-4 of SKUAST- Kashmir [12], which includes wheat straw 300 kg; rice bran 50 kg; chicken manure 150 kg; urea 5 kg; potash 2 kg; molasses 12.5 kg and gypsum 15 kg) were used. Prior to inoculating the pathogen (*T. harzianum*), each fungicidal concentration was combined with the casing mixture at a rate of 100 mL/kg. Layer spawning was performed at the rate of 0.6%. Casing was prepared by laying a 2–3 cm thick layer (about 2 kg) of casing material (soil and peat, 2:1) on spawn run composting mixture in 10 kg polybags (18 × 24 cm). *T. harizianum* was inoculated by spraying spore suspension (1 × 10^4^ mL^−1^ of sterile water) from a 5-day-old culture over casing material at a rate of 20 mL/bag. After casing, the poly bags were kept at 23 ± 2 °C in an incubation room at 75–80% relative humidity (RH). From the sixth day onwards, the temperature and the relative humidity were maintained at 10–20 °C and 75%, respectively. Each treatment characterized by a single bag was simulated thrice. The control received no fungicidal treatment with or without pathogen inoculation. Mushrooms were harvested in four flushes. The data on percent disease intensity and the total mushroom weight and yield per quintal compost were recorded for 1.5 months.

Disease control was calculated by using the formula:

(2) $$\mathrm{Disease}\;\mathrm{Control}=\frac{C-T}{C}\times 100\;$$

where *T* = % disease in Treatment (concentration); *C* = % disease in check I (Infested-untreated). (Check I and Check II were run without any concentrations)

### 2.4. Yield Data and Statistical Analysis

The experiment was laid in completely randomized design plan (CD at 5%) at 06 treatments for non-systemic fungicides and 05 treatments for systemic fungicides with each at 05 levels. Data on yield, including the quantity of fruiting bodies and their weight (g) per bag per treatment were recorded regularly, up to 40 days. Yield data were represented in kilograms of mushroom per 100 kg of compost material. Statistical analysis (Tables S1 and S2) of non-systemic and systemic fungicides data obtained from in vitro and in vivo were performed using the R Studio Desktop (version 4.2.1) [13].

## 3. Results

In the present study, the isolated pathogen of green mold disease of button mushroom was found to be caused by *T. harizianum*. The fungal characteristics, *viz*., mycelial color and growth, shape, size, color and septation of hyphae, conidiophores, conidia and phialides were compared with standard descriptions of *Trichoderma* sps given by Rifai (1969) [9]. The pathogen was reconfirmed from ITCC, IARI, New Delhi, India with accession No. 11148.19.

### 3.1. Effect of Non-Systemic Fungitoxicants

#### 3.1.1. Effect of Non-Systemic Fungitoxicants under In Vitro Conditions

**Figure 1:**
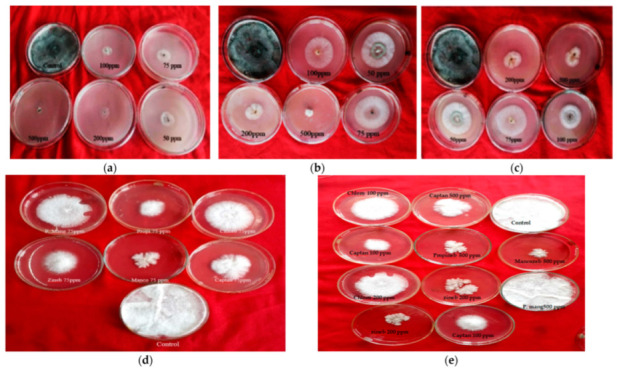
Effect of non-systemic fungitoxicants on radial mycelial growth of *T. harzianum* and *A. bisporus*: (**a**) Chlorothalonil fungitoxicant against *T. harzianum*; (**b**) Prochloraz manganese fungitoxicant against *T. harzianum*; (**c**) Captan fungitoxicant against *T. harzianum*; (**d**,**e**) P. mang (=Prochloraz manganese), Propi (=propineb), Chloro (=chlorothalonil), zineb, Manco (=mancozeb) and captan fungitoxicant against *A. bisporus*.

Six non-systemic fungitoxicants, namely chlorothalonil, mancozeb, captan, prochlo-raz manganese, zineb and propineb, were tested for inhibitory action on pathogen (*T. harzianum*) and host (*A. bisporus*) mycelium at 50, 75, 100, 200 and 500 g mL^−1^ doses using the poisoned food technique (Figure 1).

#### 3.1.2. Effect of Non-Systemic Fungitoxicants on Mycelial Growth of Pathogen (*T. Harzianum*)

**Figure 2:**
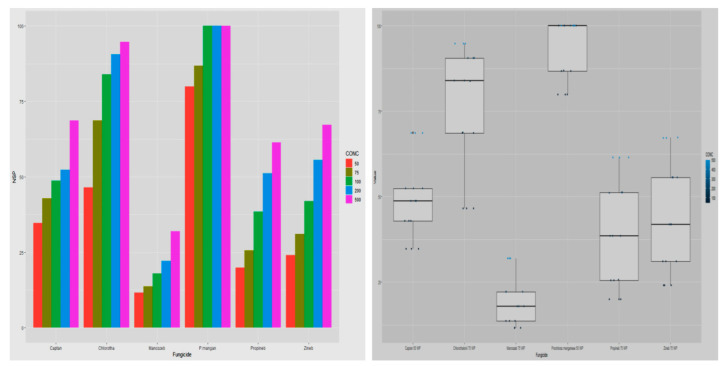
Comparative bar chart (**left**) and Boxplots (**right**) describe the effect of non-systemic fungitoxicants under in vitro conditions against *T. harzianum* mycelium.

Prohloraz manganese was found most efficient, inhibiting growth by 93.40 percent, trailed by chlorothalonil and captan displaying inhibition percentage of 76.87 and 49.44, respectively. Among different non-systemic fungicides mancozeb showed least inhibitory percentage (19.40%) against the test pathogen (Table 2; Figure 2). There was also a strong association between fungicides and their concentrations. The study also indicated that increasing fungicide concentration led to a proportionate increase in percent growth inhibition of the test pathogen, with highest overall inhibition of 70.64 percent obtained at the highest concentration of 500 μg mL^−1^; whereas an overall growth inhibition of 36.09 percent was obtained at 50 μg mL^−1^ concentration.

The maximum and minimum inhibition percentages of all evaluated non-systemic fungicides were achieved at concentration 500 and 50 μg mL^−1^, respectively. The development of the test pathogen was fully inhibited by prochloraz manganese at 100 μg mL^−1^ and higher concentrations. After prochloraz manganese chemical, the next best fungicide showing 94.73 and 90.56 percent growth inhibition was chlorothalonil at a concentration of 500 μg mL^−1^ and 200 μg mL^−1^, respectively. However, fungitoxicants mancozeb proved to be least effective at 50 μg mL^−1^ with minimal growth inhibition of just 11.57 percent.

#### 3.1.3. Effect of Non-Systemic Fungitoxicants on Mycelial Growth of Host (*A. bisporus*)

**Figure 3:**
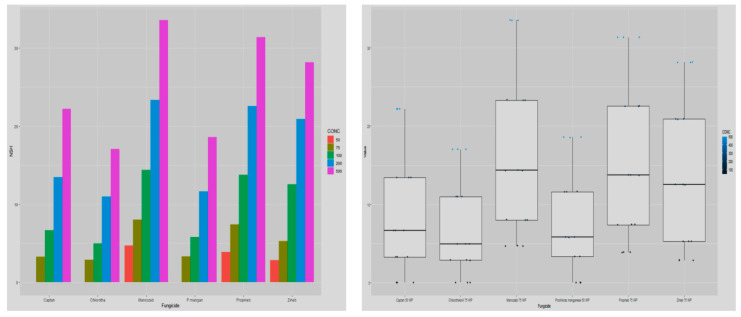
Comparative bar chart (**left**) and boxplots (**right**) designate the effect of non-systemic fungi toxicants under in vitro conditions on host *A. bisporus* mycelium.

Results (Table 3; Figure 3) showed that the inhibitory effects of the test fungicides on the host mycelium differed significantly. Overall, chlorothalonil reported minimum growth inhibition of 7.16 percent followed by prochloraz manganese and captan with inhibition percentage of 7.85 and 9.10 percent, respectively; whereas mancozeb displayed maximum growth inhibition of 16.77 percent of host mycelium. Research findings also revealed that there was a significant interaction between fungicides and their concentrations, with an increase in the concentration of fungicides the percentage of growth inhibition also increased. Minimum mean growth inhibition (1.89%) was recorded at 50 μg mL^−1^ whereas the highest (25.12%) was attained at 500 μg mL^−1^. At concentration 50 μg mL^−1^, prochloraz manganese, chlorothalonil and captan showed no growth inhibition of the host mycelium. However, at concentration 50 μg mL^−1^ fungicide zineb, propineb and mancozeb exhibited inhibition percentage of 2.84, 3.86 and 4.69, respectively. The next least inhibitory fungicides with respective concentration were chlorothalonil, captan, prochloraz manganese at 75 μg mL^−1^ with inhibition percentage of 2.88, 3.27 and 3.32, followed by chlorothalonil, zineb prochloraz manganese, captan with inhibition percentage 4.94, 5.28, 5.80, 6.66 at concentration 100, 75, 100 and 100 μg mL^−1^, respectively. Highest inhibition percentage of 33.52 and 31.34 of test fungal mycelium growth was displayed by mancozeb and propineb, respectively, at 500 μg mL^−1^.

#### 3.1.4. Effect of Non-Systemic Fungitoxicants under In Vivo Conditions

##### Effect of on Disease Development

**Figure 4:**
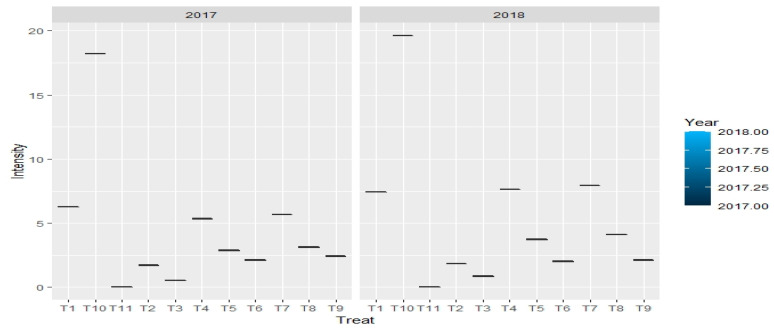
Impact of non-systemic fungitoxicants in casing on percent intensity of green mold disease of button mushroom, where T1 = Prochloraz manganese 0.05% conc., T10 = Check I(infested-untreated), T11 = Check II (uninfested-untreated), T2 = Prochloraz manganese 0.1% conc., T3 = Prochloraz manganese 0.2% conc., T4 = Chlorothalonil 0.05% conc., T5 = Chlorothalonil 0.1% conc., T6 = Chlorothalonil 0.2% conc., T7 = Captan 0.05% conc., T8 = Captan 0.1% conc., T9 = Captan 0.2% conc.

When compared to pathogen-infested and untreated check-I, the findings mentioned in Table 4 and Figure 4, demonstrated that all fungicidal treatments lowered the percent disease intensity. Compared to a green mold intensity of 18.93 percent obtained in pathogen infested with no fungicidal treatment (check-I). Application of prochloraz manganese at 0.1–0.2% or chlorothalonil at 0.2% resulted in disease control of 89.06–96.30%. Captan at 0.2%, chlorothalonil at 0.1% and captan at 0.1% were the subsequent efficient fungicides with respective concentrations displaying green mold disease intensity of 2.27–3.62 percent with a disease control of 80.87–88.00 percent. Fungitoxicant treatment of prochloraz manganese and captan at 0.05% were found least efficient by exhibiting green mold disease intensity of 6.80–6.85 with a disease control of only 63.81–64.07 percent.

##### Impact of Non-Systemic Fungitoxicants on Yield of Button Mushroom

**Figure 5:**
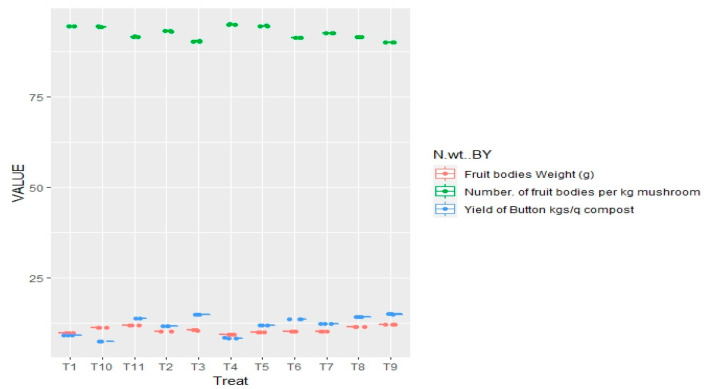
Boxplot designates the effect of non-systemic fungitoxicants on *T. harzianum* infested casing pooled during spring 2017 and 2018 on the number and weight of fruit bodies and button yield, where T1 = Prochloraz manganese 0.05% conc., T10 = Check I(infested-untreated), T11 = Check II(uninfested-untreated), T2 = Prochloraz manganese 0.1% conc., T3 = Prochloraz manganese 0.2% conc., T4 = Chlorothalonil 0.05% conc., T5 = Chlorothalonil 0.1% conc., T6 = Chlorothalonil 0.2% conc., T7 = Captan 0.05% conc., T8 = Captan 0.1% conc., T9 = Captan 0.2% conc.

Among different non-systemic fungicides that were used for study, captan showed a minimum number (90.08) of fruit bodies per kg of mushrooms at a concentration of 0.2%, trailed by prochloraz manganese and chlorothalonil at a concentration of 0.2% and captan at a concentration of 0.1% yielding 90.46–91.68 fruit bodies per kg of mushroom, whereas, 94.48 and 91.73 fruit bodies per kg of mushroom were recorded in infested-untreated and uninfested untreated, respectively, as illustrated in Table 5 and Figure 5. With distinct fungicidal treatments, the average fruit-body weight also differed significantly. The maximum (11.54–12.16 g) average single fruit body weight was obtained from polybags with treatment of captan, which was close to that obtained in uninfested-untreated control (11.86 g) at concentrations of 0.1 and 0.2 percent. The next best treatment was prochloraz manganese and chlorothalonil at concentration 0.20%, with mean fruiting weight of 10.52 and 10.24 g, respectively, compared to 11.32 g for the infested-untreated control.

By the application of non-systemic fungitoxicants, it was observed that mushroom crop yield from per quintal compost also grew dramatically. Highest button yield for treatments receiving captan (0.10–0.20%) and prochloraz manganese (0.20%) was 14.18–14.96 kg/qt compost, compared to 7.46 and 13.78 kg/qt compost obtained from controls that were infested-untreated and uninfested-untreated. The next best treatments were chlorothalonil (0.10%) or captan (0.05%) with mean yield of (12.33–13.52) kg/qt compost. The least successful fungicides at a concentration of 0.05 percent were both prochloraz manganese and chlorothalonil. From the table, it is evident, with an increase in concentration of particular fungicides, button yield also increased, e.g., prochloraz manganese at 0.2% concentration give 14.88 kg/qt compost.

##### Effect on Sporophore

**Figure 6:**
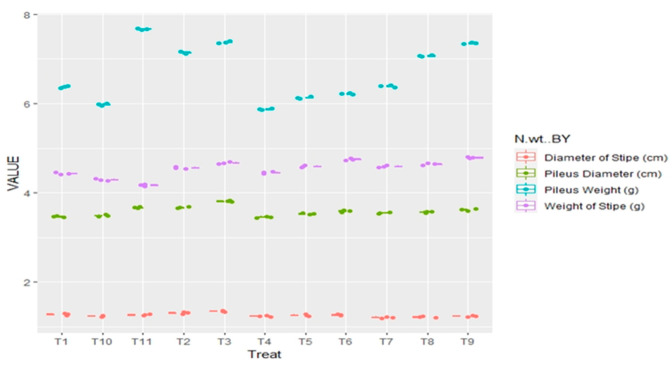
Boxplots for impact of non-systemic fungicides on *T. harzianum* infested casing pooled during spring 2017 and 2018 on quality parameters of *A. bisporus*, where T1 = Prochloraz manganese 0.05% conc., T10 = Check I(infested-untreated), T11 = Check II(uninfested-untreated), T2 = Prochloraz manganese 0.1% conc., T3 = Prochloraz manganese 0.2% conc., T4 = Chlorothalonil 0.05% conc., T5 = Chlorothalonil 0.1% conc., T6 = Chlorothalonil 0.2% conc., T7 = Captan 0.05% conc., T8 = Captan 0.1% conc., T9 = Captan 0.2% conc.

The quality characters of sporophores, *viz*., weight and diameter of pileus and stipe were also significantly affected by non-systemic fungicidal applications on infested casings (Table 6; Figure 6).

- **Pileus weight:** Treatment with prochloraz manganese at concentration 0.20% produced pileus with maximum weight of 7.37 g compared to that of the infested-untreated control (5.98 g). The next best treatment was captan followed by prochloraz manganese at concentration 0.20 and 0.10% with pileus weight of 7.35 and 7.14 g, respectively.
- **Pileus diameter:** Among the various fungicidal treatments, the largest pileus diameter of 3.81 cm was measured from polybags with treatment of prochloraz at concentration 0.20%, followed by prochloraz manganese (3.67 cm) at concentration 0.10 which was statistically equal to Check II (uninfested-untreated). The next best treatments were captan and chlorothalonil at concentration 0.20% with pileus diameter of 3.62 and 3.59 cm, respectively.
- **Stipe weight:** Treatment with captan at concentration 0.20% produced stripes with maximum weight of 4.79 g compared to that in infested-untreated control (4.29 g). The subsequent efficient treatments for producing buttons with maximum stripe weight are, chlorothalonil, prochloraz manganese 50 WP and captan 50 WP at concentration 0.20, 0.20 and 0.10, respectively. For uninfested-untreated control, the mean stipe weight was 4.17 cm.
- **Stipe diameter:** Prochloraz manganese (0.20%) treatment produced mushrooms with highest stipe diameter of 1.35 cm followed by prochloraz manganese at concentration 0.10% with a stipe diameter of 1.31 cm compared to 1.22 cm obtained in infested-untreated control.

### 3.2. Effect of Systemic Fungitoxicants

#### 3.2.1. Effect of Systemic Fungitoxicants under In Vitro Conditions

**Figure 7:**
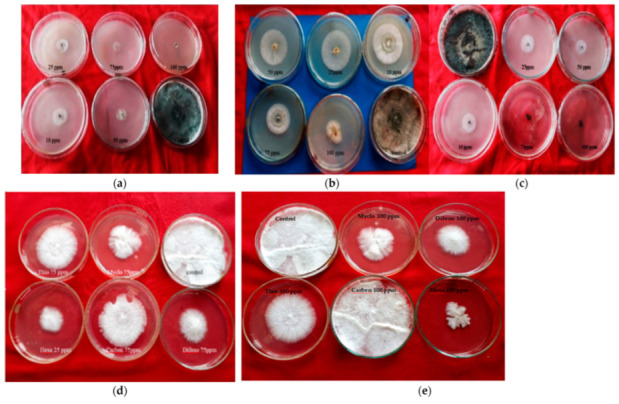
Effect of systemic fungitoxicants on radial mycelial growth of *T. harzianum* and *A. bisporus*: (**a**) thiophanate methyl against *T. harzianum*; (**b**) myclobutanil against *T. harzianum;* (**c**) carbendazim against *T. harzianum;* (**d**,**e**) Thio (= thiophanate methyl), Myclo (= myclobutanil), Hexa (= hexaconazole), Carben (= carbendazim) and Difeno (= difenoconazole) fungitoxicant against *A. bisporus*.

Five systemic fungitoxicants, namely myclobutanil, carbendazim, hexaconazole, methyl thiophanate and difenoconazole, were evaluated for their inhibitory effects on the pathogen (*T. harzianum)* and host (*A. bisporus*) mycelium at different concentrations of 10, 25, 50, 75 and 100 μg mL^−1^ by poisoned food technique (Figure 7).

#### 3.2.2. Effect of Systemic Fungitoxicants on Mycelial Growth of Pathogen (*T. harzianum*)

**Figure 8:**
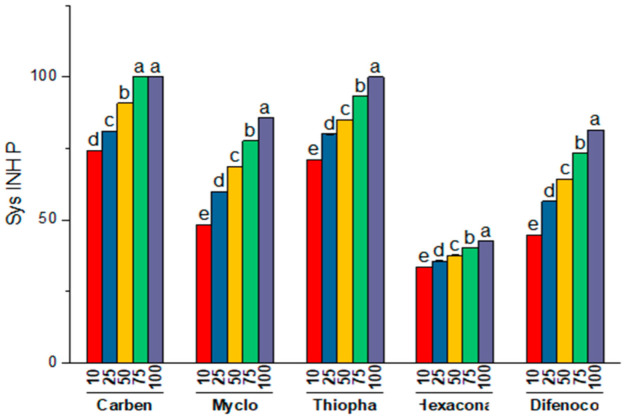
Comparative bar chart (left) designates the effect of systemic fungitoxicants under in vitro conditions against *T. harzianum* mycelium, where Carben = Carbendazim 50 WP, Myclo = Myclobutanil 10 WP, Thiopha = Thiophanate methyl 70 WP, Hexacona = Hexaconazole 5 EC, Difenoc = Difenoconazole 25 EC, Sys INHP = mycelial inhibition of Pathogen.

On average, carbendazim was most efficient inhibiting mycelial growth of 89.22 percent trailed by thiophanate methyl and myclobutanil displaying inhibition percentage of 85.89 and 68.07, respectively. It is obvious from Table 7 and Figure 8, as concentrations increased, the percent inhibition compared to the control also increased considerably, with maximal and minimal growth inhibition of 81.94 and 54.38 percent at concentration 100 and 10 μg mL^−1^, respectively. There was also a substantial relationship between fungicides with its concentrations. Thiophanate methyl and carbendazim at concentration 75 and 100 μg mL^−1^, showed maximum growth inhibition of 100 percent of test pathogen. Thiophanate methyl and carbendazim were the next efficient fungitoxicants at concentration 75 and 50 μg mL^−1^ and exhibited mycelial growth inhibition of 93.32 and 90.81 percent, respectively.

#### 3.2.3. Effect of Systemic Fungitoxicants on Mycelial Growth of Host (*A. bisporus*)

**Figure 9:**
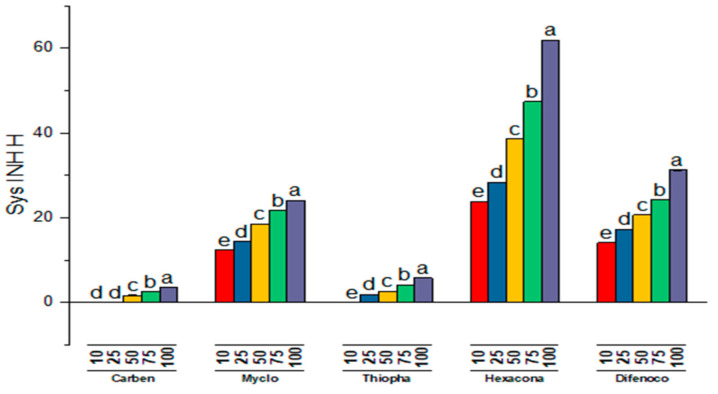
Comparative bar chart designates the effect of systemic fungitoxicants under in vitro conditions on host *A. bisporus* mycelium, where Carben = Carbendazim 50 WP, Myclo = Myclobutanil 10 WP, Thiopha = Thiophanate methyl 70 WP, Hexacona = Hexaconazole 5 EC, Difenoc = Difenoconazole 25 EC, Sys INHH = mycelial inhibition of host.

The inhibitory action of all the fungicides on host mycelium when evaluated differed substantially (Table 8; Figure 9). Carbendazim recorded the lowest growth inhibition (1.56%) of *A. bisporus* trailed by thiophanate methyl (2.90%) in overall comparison. Myclobutanil was the next least inhibitory (18.21%) to the *A. bisporus* mycelium, whereas difenoconazole and hexaconazole showed highest growth inhibition (21.48–39.99%). The mycelial inhibition was observed to rise continuously when the fungicide concentration was increased. Carbendazim showed no inhibition of the host fungus at up to 25 μg mL^−1^ and thiophanate methyl at 10 μg mL^−1^; however, at concentrations 50–100 and 25–50 µg mL^−1^ these were least inhibitory by displaying inhibitory percentages of 1.72–3.57, respectively. Fungitoxicants including hexaconazole, difenoconazole and myclobutanil were highly suppressive against *A. bisporus.* At a concentration of 100 µg ml^−1^ fungicides, viz., hexaconazole, difenoconazole and myclobutanil, exhibited inhibitory percentages of 24.13, 31.19 and 61.90 to mycelial growth of mushroom.

#### 3.2.4. Effect of Systemic Fungitoxicants under In Vivo Conditions

##### Effect on Green Mold Disease Development

**Figure 10:**
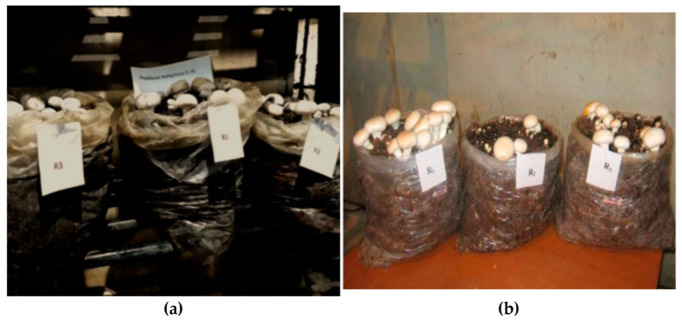

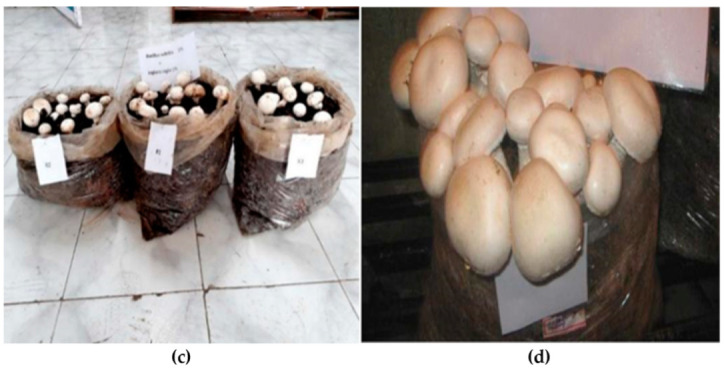
Effect of fungitoxicants under in vivo conditions: (**a**) experiment plan of layout; (**b**) prochloraz manganese treatment; (**c**) carbendazim treatment; (**d**) casing soil (uninfested).

**Figure 11:**
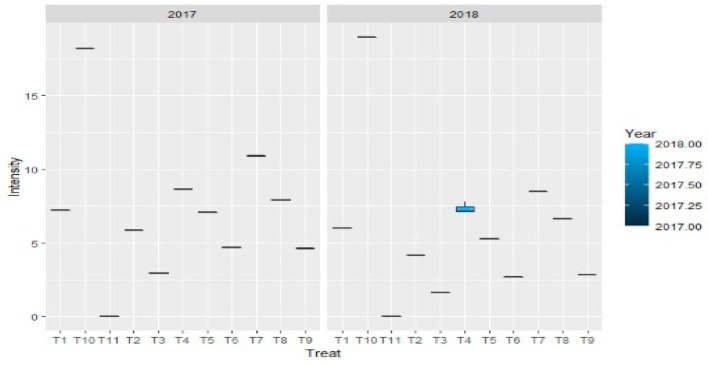
Boxplots depict the impact of systemic fungitoxicants in casing on percent intensity of green mold disease, where T1 = Carbendazim 0.025% conc., T10 = Check I (infested-untreated), T11 = Check II (uninfested-untreated), T2 = Carbendazim 0.05% conc., T3 = Carbendazim 0.1% conc., T4 = Thiophanate methyl 0.025% conc., T5 = Thiophanate methyl 0.05% conc., T6 = Thiophanate methyl 0.1% conc., T7 = Myclobutanil 0.025% conc., T8 = Myclobutanil 0.05% conc., T9 = Myclobutanil 0.1% conc.

Table 9 and Figure 10 revealed the effect of systemic fungitoxicant used in casing soil on green mold disease of mushroom. Findings revealed that all fungitoxicants decreased the percent disease inhibition when compared with pathogen infested-untreated check-I. Compared to disease intensity of 18.56 percent in pathogen uninfested-untreated check-I, the infection was decreased to 2.29–3.69 percent while using carbendazim 50 WP or thiophanate methyl 70 WP at concentration 0.1 percent, resulting in 80.11–87.66 percent disease control. The next best treatments with green mold intensity of 3.73–6.18 percent with a disease control of 66.70–79.90 percent were myclobutanil at 0.1 percent or carbendazim at 0.05 percent concentration or thiophanate methyl at 0.05 percent concentration. Myclobutanil at 0.025 and thiophanate methyl and carbendazim at 0.025 percent with a disease control of 47.73–64.38 percent were the least successful fungicidal treatment with green mold intensity of 6.61–9.70 percent. However, in the pathogen uninfested-untreated check-II, a green mold-free crop without any disease severity was obtained (Figure 11).

##### Impact of Systemic Fungitoxicants on Yield

**Figure 12:**
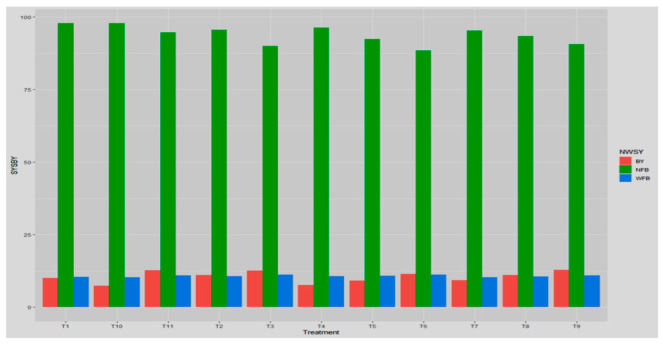
Comparative bar chart designates the effect of systemic fungitoxicants on *T. harzianum* infested casing pooled during spring 2017 and 2018 on the number and weight of fruit bodies and button yield, where T1 = Carbendazim 0.025% conc., T10 = Check I (infested-untreated), T11 = Check II (uninfested-untreated), T2 = Carbendazim 0.05% conc., T3 = Carbendazim 0.1% conc., T4 = Thiophanate methyl 0.025% conc., T5 = Thiophanate methyl 0.05% conc., T6 = Thiophanate methyl 0.1% conc., T7 = Myclobutanil 0.025% conc., T8 = Myclobutanil 0.05% conc., T9 = Myclobutanil 0.1% conc.

Significant impacts on yield and yield parameters such as weight and number of fruiting bodies were seen in the fungicidal application on pathogen infested casings. From Table 10 and Figure 12, it is evident that the least number of fruit bodies per kg of mushroom are 88.50–90.07 and were reported in treatments receiving thiophanate methyl or carbendazim each at 0.1 percent compared to uninfested-untreated controls (93.47). The next best fungicides were myclobutanil (0.1%) and thiophanate methyl (0.05%), producing 90.67–92.43 of fruit bodies. However, yield was not greatly affected by thiophanate methyl, carbendazim when applied at 0.025 percent concentration.

Applying systemic fungicides to *T. harzianum*-infested casings, the average weight of fruit-bodies was significantly affected. Thiophanate methyl (0.1% concentration) and carbendazim (0.025% concentration) produced 10.44–11.26 g of average fruiting body weight, which increased from 10.33 g obtained from infested-untreated polybags. Fruiting bodies weighing 10.56 g were obtained in uninfested-untreated control. The next best fungicide treatments were carbendazim 50 WP (0.1%) and myclobutanil (0.1%) producing fruit bodies of 10.96–11.21 g weight.

Button mushroom yield (kg per quintal compost) has been increased significantly by application of systemic fungicide. Application of fungicides, myclobutanil and carbendazim both displayed a maximum yield of 12.63 and 12.87 kg q^−1^ compost at 0.1 percent concentration that was as good as that obtained in the uninfested control (11.08 kg q^−1^ compost). The same was found in the systemic fungitoxicants, yield increase with an increase in fungitoxicant concentration of particular fungitoxicant, e.g., in carbendazim the highest yield was achieved at 0.1% concentration, which gave 12.63 kg q^−1^ compost.

##### Effect on Sporophores

**Figure 13:**
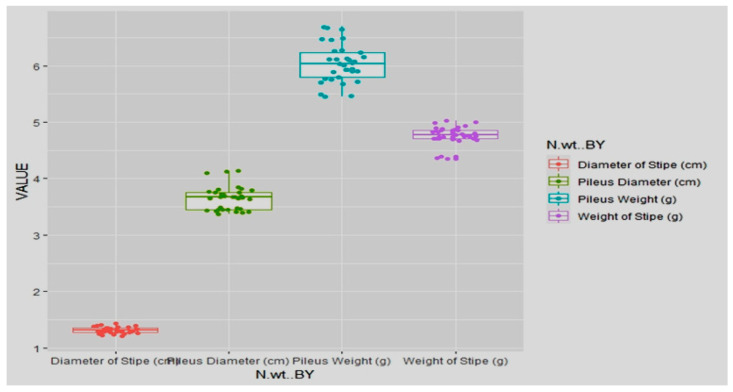
Boxplots for impact of systemic fungicides on *T. harzianum* infested casing pooled during spring 2017 and 2018 on quality parameters of *A. bisporus*.

The fungitoxicant impact on the infested casing has greatly afflicted the quality parameters of sporophore, such as diameter of pileus and weight of stipe, although there was no substantial change in the pileus weight and stipe diameter (Table 11, Figure 13).

- **Weight of pileus:** The pileus weight of *A. bisporus* sporophores did not change considerably after fungicidal treatments in pathogen-infested casing.
- **Pileus diameter:** The maximum pileus diameter of 3.82 cm was recorded in polybags receiving myclobutanil (0.1%) fungitoxicant treatment. Carbendazim (0.1%) or myclobutanil (0.05%) or thiophanate methyl (0.05%) applications were the next best treatments that produced 3.67–3.73 cm of pileus diameter, parallel to those attained in infested-untreated controls. However, none of the fungitoxicant applications developed a pileus diameter of 4.12 cm like that of the uninfested-untreated control.
- **Stipe weight:** Weight of stripe enlarged significantly to 4.91–5.01 g at 0.1 percent in treatments receiving carbendazim or myclobutanil, compared to 4.37 g in infested-untreated control and 4.69 g in uninfested-untreated control. Thiophanate methyl (0.1%), myclobutanil (0.05%) or carbendazim (0.05%), yielding a mean stipe weight of 4.83–4.87 g, were the next best treatments.
- **Stipe diameter:** The fungicidal treatment had no discernible effect on the stipe diameter of *A. bisporus* sporophores.

## 4. Discussion

Usage of non-chemical (fungicide) ingredients such as botanicals and bio-control agents are ideally necessary for the successful management of green mold disease in mushroom production houses. It is also possible to use certain fungicides to control the disease economically. All these applications and amendments are, however, intended to verify or inhibit the proliferation and growth of *T. harzianum* with no or minimal impact on host mycelium (*A. bisporus*). Under in vitro and in vivo conditions, different fungicides were tested against both *T. harzianum* and *A. bisporus* in order to choose the most efficient ones regarding the disease’s restraint. Maximum reticence under in vitro conditions against *T. harzianum* was exhibited by prochloraz manganese, followed by chlorothalonil, and were least effective against *A. bisporus* growth. Among six in vitro fungitoxicants tested, chlorothalonil, prochloraz manganese and captan were further assessed in a mushroom house for in vivo efficacy. In contrast to other fungicides, the selected fungitoxicants expressed partial restraint of mycelium of mushrooms but maximum pathogen inhibition. Among systemic fungitoxicants, carbendazim, thiophante methyl and myclobutanil were highly inhibitory to *T. harzianum* and were less inhibitory to *A. bisporus* mycelium growth; whereas the least inhibitory fungicide against the pathogen was hexaconazole under in vitro conditions. Carbendazim displayed a lower degree of mushroom inhibition, suggesting that *A. bisporus* is marginally resistant to this fungitoxicant. Our results were in limited covenant with Thapa and Seth [14], and demonstrated carbendazim as the best fungicide against *Trichoderma* without affecting mushroom growth. By application of carbendazim or thiophanate methyl at concentration 0.1 percent, the infection was reduced to 2.29–3.69 percent with disease control of 80.11–87.66 percent. Again, the influence of systemic fungitoxicants does not have much impact on sporophore quality parameter. Similarly, several other workers have also documented the inhibitory effects of these fungicides, thus these results were in agreement with the present findings [15,16,17,18,19,20,21]. Different systemic and non-systemic fungicides such as carbendazim, bitertanol, hexaconzole, captan and mancozeb were evaluated against the pathogen [15]. The findings showed that the highest mycelial mean inhibition of *T. harzianum* was observed in carbendazim followed by bitertanol, captan, hexaconzole and at least mancozeb, which strongly supports the present findings. Prochloraz manganese is the officially recommended fungicide in the mushroom industry. The aggressive growth of *Trichoderma* isolates has been found to be inhibited without affecting the growth of *A. bisporus* by prochloraz manganese, as it interferes with the demethylation step in ergosterol biosynthesis [16,19]. Several fungicides for the control of green mold disease were also evaluated [19], including prochloraz, prochloraz + carbendazim and thiabendazole and even environ (a commercial disinfectant). In vitro evaluation of various fungicides against *Trichoderma* spp. isolated from button mushroom were studied and carbendazim was proved most effective [22]. *Trichoderma* growth was also inhibited by benomyl, prochloraz manganese and imazalil, different fungicides were considered by IC_50_ values > 2 ppm for both thermophilic fungi and *A. bisporus*. Prochloraz, flusilazol and benomyl had partial effects on *A. bisporus* growth and development between the concentration of 0 and 2 ppm but were found extra lethal for thermophilic fungi [23,24].

With the in vivo assessment of this pathogen, it would possible to determine the effectiveness of every aspect of disease management. The in vivo evaluation of fungitoxicants showed that by using prochloraz manganese at 0.10–0.2 percent or chlorothalonil at 0.2 percent, the disease was reduced to 0.70–2.07 percent, showing disease control of 89.06–96.30 percent. Due to the impact of non-systemic fungitoxicants, there is no influence on the quality parameters of sporophores, carbendazim and thiophanate methyl (systemic) or prochloraz manganese and chlorothalonil (non-systemic) were successful in controlling green mold disease with a concomitant increase in the yield of button mushrooms incorporated into pathogen-infested casing soil. Furthermore, growth inhibition of *Pleurotus* sp. and *T. viride* increased with an increase in concentration of various fungicides [25]. The most effective fungicide against green mold treatment was carbendazim [26], in addition to this fungicide bavistin and sporgon (prochloraz manganese) were also efficient in completely suppressing the mycelial development of *Trichoderma* sp. when added to an agar medium at 5 ppm and 10 ppm, respectively, and, hence, supports our present findings. Other researchers have also demonstrated the inclusion of efficient fungicides, bio control agents or botanicals for the control of green mold disease of button mushrooms [27,28,29]. The efficacy of prochloraz manganese and chlorothalonil against *T. Harzianum* edible fungi mold was also reported earlier by some workers [16,18,19,21].

## 5. Conclusions

The present finding on evaluation of non-systemic and systemic fungicides revealed the promising effects in reducing the infection of green mold. At 100 μg mL^−1^ concentration, carbendazim and thiophante methyl fully inhibited the growth of the pathogen under in vitro and in vivo. Among non-systemic prochloraz, manganese was found to be highly effective in reducing the disease. Carbendazim at 0.1% and prochloraz manganese at 0.2% provided an efficient control of green mold infection of white button mushroom. However, biological control of disease management, an alternative to fungicides, is also highly recommended and encouraged.

## Figures and Tables

**Table 1:** Fungitoxicants evaluated against the green mold pathogen of white button mushroom (*A. bisporus*).

Common Name	Manufacture Name	Trade Name
(a) Non-systemic		
Prochloraz Manganese	FMC company	Sportek 50 WP
Chlorothalonil	M/S Syngenta India Ltd.	Kavach 75 WP
Mancozeb	M/S Dow Agro Science Mumbai	Dithane M 45
Captan	M/S Rallis India Ltd.	Captaf 50 WP
Propineb	M/S Bayer Crop Science Ltd.	Antracol 70 WP
Zineb	M/S Indofil Industries Ltd.	Indofil Z-78 (75 WP)
(b) Systemic		
Carbendazim	Crystal Crop Protection Pvt Limited	Bavistin 50 WP
Thiophanate methyl	Mahashantam Pesticides Private limited	Topsin M 70 WP
Difenoconazole	M/S Syngenta India Ltd.	Score 25 EC
Myclobutanil	M/S Dow Agro Science Mumbai Ltd.	Systhane 10 WP
Hexaconazole	M/S Rallis India Ltd.	Contaf 5 EC

**Table 2:** Effect of non-systemic fungitoxicants under in vitro conditions against *T. harzianum* mycelium.

	Conc. (µg mL^−1^)	Inhibition of Growth over Control (%) *					Mean
Fungicide		50	75	100	200	500	
Prochloraz manganese 50 WP		79.86 (63.30)	86.80 (68.96)	100.00 (89.42)	100.00 (89.42)	100.00 (89.42)	93.40 (80.10) ^a^
Chlorothalonil 75 WP		46.52 (42.98)	68.66 (55.93)	83.88 (66.25)	90.56 (72.07)	94.73 (76.69)	76.87 (62.78) ^b^
Mancozeb 75 WP		11.57 (19.87)	13.63 (21.65)	17.90 (25.01)	22.06 (28.00)	31.88 (34.36)	19.40 (25.78) ^f^
Captan 50 WP		34.68 (36.06)	42.85 (40.86)	48.70 (44.23)	52.35 (46.32)	68.65 (55.92)	49.44 (44.68) ^c^
Propineb 75 WP		19.94 (26.51)	25.52 (30.33)	38.48 (38.32)	51.12 (45.62)	61.43 (51.58)	39.29 (38.47) ^e^
Zineb 75 WP		23.98 (29.30)	31.01 (33.82)	41.88 (40.31)	55.60 (48.19)	67.19 (55.03)	43.93 (41.33) ^d^
Mean		36.09 (36.33) ^e^	44.74 (41.92) ^d^	55.14 (50.59) ^c^	61.94 (54.93) ^b^	70.64 (60.50) ^a^	
		S.E±			CD (*p* ≤ 0.05)		
Fungicides		(0.074)			(0.020)		
Concentration		(0.067)			(0.019)		
Fungicide × Concentration		(0.165)			(0.047)		

* Mean of three replications; values in parentheses are angular transformed values; means followed by similar letter are statistically identical. For statistical analysis, the values of 100 were taken as 99.99.

**Table 3:** Effect of non-systemic fungitoxicants under in vitro conditions on host *A. bisporus* mycelium.

	Conc. (µg mL^−1^)	Inhibition of Growth over Control (%) *					Mean
Fungitoxicant		50	75	100	200	500	
Prochloraz manganese 50 WP		0.0 (0.57)	3.32 (10.49)	5.80 (13.93)	11.61 (19.91)	18.56 (25.50)	7.85 (14.08) ^b^
Chlorothalonil 75 WP		0.0 (0.57)	2.88 (9.76)	4.94 (12.83)	10.98 (19.34)	17.03 (24.36)	7.16 (13.37) ^a^
Mancozeb 75 WP		4.69 (12.50)	8.01 (16.43)	14.34 (22.24)	23.31 (28.85)	33.52 (35.36)	16.77 (23.08) ^f^
Captan 50 WP		0.0 (0.57)	3.27 (10.41)	6.66 (14.95)	13.42 (21.48)	22.19 (28.09)	9.10 (15.1) ^c^
Propineb 75 WP		3.86 (11.32)	7.41 (15.79)	13.74 (21.74)	22.54 (28.33)	31.34 (34.03)	15.77 (22.24) ^e^
Zineb 75 WP		2.84 (9.69)	5.28 (13.27)	12.51 (20.70)	20.89 (27.18)	28.13 (32.01)	14.24 (20.57) ^d^
Mean		1.89 (5.87) ^a^	5.20 (12.69) ^b^	9.66 (17.73) ^c^	17.12 (24.18) ^d^	25.12 (29.89) ^e^	
		S.E±			CD (*p* ≤ 0.05)		
Fungicides		(0.005)			(0.015)		
Concentration		(0.004)			(0.013)		
Fungicide × Concentration		(0.012)			(0.034)		

* Mean of each * each treatment replicated thrice; values in parentheses are angular transformed values; means followed by similar letter are statistically identical. For statistical analysis the values of 0 were taken as 0.1.

**Table 4:** Impact of non-systemic fungitoxicants in casing on percent intensity of green mold disease of button mushroom.

Treatments	Concentration	Spring 2017 *	Spring 2018 *	Pool *	Disease Control
Prochloraz manganese 50 WP	0.05%	6.28 (2.69)	7.42 (2.90)	6.85 (2.79) ^b^	63.81
	0.1%	1.72 (1.64)	1.84 (1.68)	1.78 (1.66) ^a^	90.59
	0.2%	0.54 (1.24)	0.86 (1.36)	0.70 (1.30) ^a^	96.30
Sub mean		2.84 (1.85)	3.37 (1.98)	3.11 (1.91)	
Chlorothalonil 75 WP	0.05%	5.34 (2.51)	7.63 (2.93)	6.48 (2.72) ^b^	65.76
	0.1%	2.87 (1.96)	3.73 (2.17)	3.30 (2.07) ^b^	82.56
	0.2%	2.12 (1.76)	2.02 (1.73)	2.07 (1.75) ^a^	89.06
Sub mean		3.44 (2.07)	4.46 (2.27)	3.95 (2.18)	
Captan 50 WP	0.05%	5.67 (2.58)	7.93 (2.98)	6.80 (2.78) ^b^	64.07
	0.1%	3.13 (2.03)	4.11 (2.26)	3.62 (2.14) ^b^	80.87
	0.2%	2.42 (1.84)	2.12 (1.76)	2.27 (1.80) ^a^	88.00
Sub mean		3.74 (2.15)	4.72 (2.33)	4.23 (2.24)	
Check I (infested-untreated)		18.23 (4.38)	19.63 (4.54)	18.93 (4.46) ^c^	-
Check II (uninfested-untreated)		0.0 (1.00)	0.0 (1.00)	0.0 (1.00)^a^	-
CD (*p* ≤ 0.05)					
Treatment combination		0.0091	0.0086	0.0060	
Control v/s rest		0.0082	0.0077	0.0054	
Fungicides		0.0198	0.0196	0.0196	
Concentration		0.0198	0.0196	0.0197	
Fungicide × Concentration		0.0342	0.0339	0.0338	

* Three replications mean; numbers * Mean of three replications; values in parenthesis are square root transformed values; similar letter following means are statistically identical.

**Table 5:** Effect of non-systemic fungicides on *T. harzianum* infested casing pooled during spring, 2017 and 2018 on the number of fruit bodies per kg mushroom, fruit bodies weight (g) and yield of button (kg/quintal compost).

Fungitoxicant	Concentration	Number of Fruit Bodies per kg Mushroom *	Fruit Bodies Weight (g) *	Yield of Button (kg/quintal Compost) *
Prochloraz manganese 50 WP	0.05%	94.63 ^g^	9.82 ^e^	9.13 ^e^
	0.1%	93.22 ^ef^	10.14 ^d^	11.67 ^cd^
	0.2%	90.46 ^ab^	10.52 ^c^	14.88 ^a^
Sub mean		92.77	10.16	11.89
Chlorothalonil 75 WP	0.05%	95.11 ^f^	9.26 ^e^	8.37 ^f^
	0.1%	94.68 ^e^	10.02 ^d^	11.83 ^cd^
	0.2%	91.36 ^c^	10.24 ^cd^	13.52 ^ab^
Sub mean		93.71	9.84	11.24
Captan 50 WP	0.05%	92.66 ^e^	10.16 ^cd^	12.33 ^c^
	0.1%	91.68 ^cd^	11.54 ^b^	14.18 ^ab^
	0.2%	90.08 ^a^	12.16 ^a^	14.96 ^a^
Sub mean		91.47	11.28	13.82
Check I (infested-untreated)		94.48 ^g^	11.32 ^c^	7.46 ^g^
Check II (uninfested-untreated)		91.73 ^cd^	11.86 ^a^	13.78 ^ab^
CD (*p* ≤ 0.05)				
Control v/s rest		0.0638	0.0333	0.0328
Fungicides		0.0336	0.0196	0.0206
Concentration		0.0336	0.0196	0.0206
Fungicide × Concentration		0.0538	0.0340	0.0357

* Three replications mean; similar letter followed by mean are statistically equal.

**Table 6:** Impact of non-systemic fungicides on *T. harzianum* infested casing pooled during spring 2017 and 2018 on quality parameters of *A. bisporus*.

Fungicide	Concentration	Pileus Weight (g) *	Pileus Diameter (cm) *	Weight of Stipe (g) *	Diameter of Stipe (cm) *
Prochloraz manganese 50 WP	0.05%	6.37 ^d^	3.47 ^f^	4.43 ^d^	1.28
	0.1%	7.14 ^c^	3.67 ^b^	4.56 ^c^	1.31
	0.2%	7.37 ^ab^	3.81 ^a^	4.67 ^b^	1.35
Sub mean		6.96	3.65	4.55	1.31
Chlorothalonil 75 WP	0.05%	5.87 ^e^	3.46 ^f^	4.45 ^d^	1.24
	0.1%	6.13 ^de^	3.53 ^e^	4.59 ^c^	1.26
	0.2%	6.22 ^de^	3.59 ^c^	4.75 ^a^	1.27
Sub mean		6.07	3.52	4.59	1.25
Captan 50 WP	0.05%	6.39 ^d^	3.55 ^d^	4.59 ^c^	1.21
	0.1%	7.07 ^c^	3.57 ^d^	4.64 ^b^	1.22
	0.2%	7.35 ^ab^	3.62 ^c^	4.79 ^a^	1.24
Sub mean		6.93	3.58	4.67	1.22
Check I (infested-untreated)		5.98 ^de^	3.49 ^f^	4.29 ^e^	1.24
Check II (uninfested-untreated)		7.67 ^a^	3.67 ^b^	4.17 ^f^	1.27
CD (*p* ≤ 0.05)					
Control v/s rest		0.0338	0.0338	0.0340	0.0338
Fungicides		0.0197	0.0198	0.0199	0.0197
Concentration		0.0197	0.0198	0.0199	0.0197
Fungicide × Concentration		0.0342	0.0343	0.0344	NS

* Three replications mean; similar letter followed by mean are statistically identical.

**Table 7:** Effect of systemic fungitoxicants under in vitro conditions against *T. harzianum* mycelium.

	Conc. (µg mL^−1^)	Inhibition of Growth over Control (%) *					Mean
Fungitoxicant		10	25	50	75	100	
Carbendazim 50 WP		74.27 (61.46)	81.04 (64.29)	90.81 (72.34)	100.00 (89.42)	100.00 (89.42)	89.22 (75.38) ^a^
Myclobutanil 10 WP		48.27 (44.11)	59.86 (50.51)	68.77 (55.96)	77.69 (61.37)	85.76 (67.80)	68.07 (55.95) ^c^
Thiophanate methyl 70 WP		71.14 (57.91)	79.96 (63.32)	85.05 (67.70)	93.32 (74.99)	100.00 (89.42)	85.89 (70.66) ^b^
Hexaconazole 5 EC		33.52 (35.36)	35.65 (36.64)	37.62 (37.81)	40.19 (39.32)	42.52 (40.68)	37.90 (37.96) ^e^
Difenoconazole 25 EC		44.73 (41.91)	56.44 (49.12)	64.35 (53.91)	73.30 (58.95)	81.42 (64.46)	64.04 (53.67) ^d^
Mean		54.38 (48.15) ^e^	62.59 (61.68) ^d^	69.32 (57.54) ^c^	76.9 (64.81) ^b^	81.94 (70.35) ^a^	
		S.E±			CD (*p* ≤ 0.05)		
Fungitoxicant		(1.41)			(0.0049)		
Concentration		(1.41)			(0.0049)		
Fungitoxicant × concentration		(3.17)			(0.0109)		

* Three replications mean; numbers in parentheses are angular transformed values; same letter following means are statistically equal. For statistical analysis the values of 100 were taken as 99.99.

**Table 8:** Effect of systemic fungitoxicants on *host A. bisporus* mycelium under in vitro conditions.

	Conc. (µg mL^−1^)	Inhibition of Growth over Control (%)*					Mean
Fungicide		10	25	50	75	100	
Carbendazim 50 WP		0.0 (0.57)	0.0 (0.57)	1.72 (7.53)	2.54 (9.16)	3.57 (10.88)	1.56 (5.74) ^a^
Myclobutanil 10 WP		12.34 (20.55)	14.41 (22.30)	18.51 (25.47)	21.70 (27.75)	24.13 (29.40)	18.21 (25.09) ^c^
Thiophanate methyl 70 WP		0.0 (0.57)	1.85 (7.81)	2.68 (9.41)	4.14 (11.73)	5.83 (13.96)	2.90 (8.69 )^b^
Hexaconazole 5 EC		23.81 (29.19)	28.34 (32.15)	38.55 (38.36)	47.37 (43.47)	61.90 (51.86)	39.99 (39.01) ^e^
Difenoconazole 25 EC		14.10 (22.04)	17.26 (24.53)	20.65 (27.01)	24.20 (29.45)	31.19 (33.93)	21.48 (27.39) ^d^
Mean		10.05 (14.58) ^e^	12.37 (17.36) ^d^	16.42 (21.56) ^c^	19.99(24.31) ^b^	25.32 (28.01)^a^	
			S.E±			CD (*p* ≤ 0.05)	
Fungitoxicant			(0.005)			(0.015)	
Concentration			(0.005)			(0.015)	
Fungitoxicant× concentration			(0.012)			(0.035)	

* Three replications mean; numbers in parentheses are angular transformed values; same letter following means are statistically equal. For statistical analysis, the values of 0 were taken as 0.1.

**Table 9:** Impact of systemic fungitoxicants in casing on percent intensity of green mold disease.

Treatments	Concentration	Spring 2017 *	Spring 2018 *	Pool *	Disease Control
Carbendazim 50 WP	0.025%	7.21 (2.86)	6.01 (2.64)	6.61(2.75) ^f^	64.38
	0.05%	5.86 (2.61)	4.17 (2.27)	5.01 (2.44) ^d^	73.00
	0.1%	2.96 (1.98)	1.63 (1.62)	2.29 (1.80) ^b^	87.66
Sub mean		5.34 (2.48)	3.93 (2.17)	4.63 (2.33)	
Thiophanate methyl 70 WP	0.025%	8.65 (3.10)	7.10 (2.84)	7.87 (2.97) ^g^	57.59
	0.05%	7.08 (2.84)	5.29 (2.50)	6.18(2.67) ^de^	66.70
	0.1%	4.70 (2.38)	2.69 (1.92)	3.69 (2.15) ^c^	80.11
Sub mean		6.81 (2.77)	5.02 (2.42)	5.91 (2.59)	
Myclobutanil 10 WP	0.025%	10.90 (3.44)	8.50 (3.08)	9.70 (3.26) ^h^	47.73
	0.05%	7.92 (2.98)	6.62 (2.76)	7.27 (2.87) ^f^	60.82
	0.1%	4.62 (2.37)	2.84 (1.95)	3.73 (2.16) ^c^	79.90
Sub mean		7.81 (2.93)	5.98 (2.59)	6.90 (2.76)	
Check I (infested-untreated)		18.16 (4.37)	18.96(4.46)	18.56(4.42) ^i^	-
Check II (uninfested-untreated)		0.0 (1.00)	0.0 (1.00)	0.0 (1.00) ^a^	-
CD (*p* ≤ 0.05)					
Treatment combination		0.0061	0.0074	0.0046	
Control v/s rest		0.0056	0.0066	0.0042	
Fungicides		0.0198	0.0192	0.0195	
Concentration		0.0198	0.0192	0.0196	
Fungicide × Concentration		0.0344	0.0333	0.0338	

* Three replications mean; numbers in parentheses are square root transformed values; similar letter following mean are statistically identical.

**Table 10:** Effect of systemic fungicides on *T. harzianum* infested casing pooled during spring 2017 and 2018 on the number of fruit bodies per kg mushroom, fruit bodies weight (g) and yield of button (kg/quintal compost).

Treatments	Concentration	Number of Fruit Bodies per kg Mushroom	Fruit Bodies Weight (g)	Yield of Button kg/qt Compost
Carbendazim 50 WP	0.025%	97.83 ^h^	10.44 ^bc^	10.05 ^c^
	0.05%	95.58 ^f^	10.66 ^b^	11.10 ^b^
	0.1%	90.07 ^b^	11.21 ^a^	12.63 ^a^
Sub mean		94.49	10.77	11.26
Thiophanate methyl 70 WP	0.025%	96.33 ^g^	10.67 ^b^	7.63 ^e^
	0.05%	92.43 ^d^	10.82 ^b^	9.12 ^d^
	0.1%	88.50 ^a^	11.26 ^a^	11.44 ^b^
Sub mean		92.42	10.91	9.39
Myclobutanil 10 WP	0.025%	95.32 ^f^	10.33 ^d^	9.33 ^d^
	0.05%	93.47 ^e^	10.56 ^b^	11.08 ^b^
	0.1%	90.67 ^bc^	10.96 ^a^	12.87 ^a^
Sub mean		93.15	10.61	11.09
Check I (infested-untreated)		95.32 ^f^	10.33 ^d^	9.33 ^d^
Check II (uninfested-untreated)		93.47 ^e^	10.56 ^b^	11.08 ^b^
CD(*p* ≤ 0.05)				
Control v/s rest		0.0638	0.0343	0.0338
Fungicides		0.0505	0.0201	0.0197
Concentration		0.0505	0.0201	0.0197
Fungicide × Concentration		0.0875	0.0349	0.0342

Three replications mean; numbers in parentheses are square root transformed values; similar letter following mean are statistically identical.

**Table 11:** Impact of systemic fungitoxicants on *T. harzianum*-infested casing pooled during spring 2017 and 2018 on quality parameters of *A. bisporus*.

Fungicide	Concentration	Pileus Weight (g) *	Pileus Diameter (cm) *	Weight of Stipe (g) *	Diameter of Stipe (cm)*
Carbendazim 50 WP	0.025%	6.13	3.47 ^d^	4.77 ^ab^	1.32
	0.05%	6.25	3.39 ^d^	4.83 ^a^	1.37
	0.1%	6.47	3.73 ^b^	5.01 ^a^	1.41
Sub mean		6.28	3.53	4.87	1.36
Thiophanate methyl 70WP	0.25%	5.47	3.45 ^d^	4.71 ^ab^	1.27
	0.05%	5.77	3.67 ^bc^	4.73 ^ab^	1.31
	0.1%	6.03	3.77 ^b^	4.87 ^a^	1.37
Sub mean		5.75	3.63	4.77	1.31
Myclobutanil 10 WP	0.025%	5.70	3.66 ^bc^	4.73 ^ab^	1.23
	0.05%	5.91	3.67 ^bc^	4.83 ^a^	1.26
	0.1%	6.10	3.82 ^b^	4.91 ^a^	1.29
Sub mean		5.90	3.71	4.82	1.26
Check I (infested-untreated)		5.93	3.43 ^d^	4.37 ^d^	1.29
Check II (uninfested-untreated)		6.67	4.12 ^a^	4.69 ^c^	1.31
CD (*p* ≤ 0.05)					
Control v/s rest		0.0338	0.0339	0.0338	0.0338
Fungicides		0.0198	0.0198	0.0199	0.0198
Concentration		0.0198	0.0198	0.0199	0.0198
Fungicide × Concentration		0.0343	0.0343	0.0344	NS

* Three replications mean; same letter following mean are statistically equal.

## Data Availability

Not applicable.

## Supplementary Materials

The following supporting information can be downloaded at: https://www.mdpi.com/article/10.3390/jof8060554/s1, Table S1: Table represents mean sum of squares of non-systemic fungicides; Table S2: Table of mean sum of squares of systemic fungitoxicants.

## References

[1] Stojchev G., Asan A., Giicin F. (1998). Some macrofungi species of European part of Turkey. Turk. J. Bot..

[2] (2021). FAOStat, 2021, FAO, Rome. http://www.fao.org/faostat.

[3] Bellettini M.B., Fiorda F.A., Maieves H.A., Teixeira G.L., Ávila S., Hornung P.S., Júnior A.M., Ribani R.H. (2019). Factors affecting mushroom *Pleurotus* spp.. Saudi J. Biol. Sci..

[4] Allaga H., Zhumakayev A., Büchner R., Kocsubé S., Szűcs A., Vágvölgyi C., Kredics L., Hatvani L. (2021). Members of the *Trichoderma harzianum* Species Complex with Mushroom Pathogenic Potential. Agronomy.

[5] Bhatt J.C., Singh R. (2000). Influence of the *Trichoderma* exudates on the growth and yield of mushroom cultivation in north plain of India. Indian Phytopathol..

[6] Castle A., Speranzini D., Rghei N., Alm G., Rinker D., Bissett J. (1998). Morphological and molecular identification of Trichoderma isolates on North American mushroom farms. Appl. Environ. Microbiol..

[7] Forer L.B., Wuest P.J., Wanger V.R. (1974). Occurrence and economic impact of fungal diseases of mushrooms in Pennsylvania. Plant Dis. Report..

[8] Holliday P. (1980). Fungus Diseases of Tropical Crops.

[9] Rifai M.A. (1969). A revision of the genus *Trichoderma*. Mycol. Pap..

[10] Nene Y.L., Thapliyal P.N. (2000). Poisoned Food Technique. Fungicides in Plant Disease Control.

[11] Mantel E.F.K., Agarwal R.K., Seth P.K. (1972). A guide to mushroom cultivation unit. Handbook of Mushrooms.

[12] Nazir A.M., Dar G.H., Ghani M.Y., Kauser S., Mughal M.N. (2011). Button Mushroom Cultivation, Horticulture Technology Mission, Annual Report SKUAST-KASHMIR.

[13] RStudio Desktop (Version 4.2.1). http://www.rstudio.com.

[14] Shah S., Nasreen S. (2013). Efficacy of fungicides against green mould (*T. harzianum*) disease in oyster mushroom cultivation. Res. J. Microbiol..

[15] Hatvani L., Kocsube S., Menczinger L., Antal Z., Szekeres A., Druzhina I.S., Kredics L., Van Greuning M. (2008). The green mould disease global threat to the cultivation of oyster mushroom (*Pleurotus ostreatus*): A review. Science and Cultivation of Edible and Medicinal Fungi: Mushroom Science XVII, Proceeding of the 17th Congress of the International Society for Mushroom Science.

[16] Danesh Y.R., Goltapeh E.M. (2007). Studies of the effects of benomyl and carbendazim on *Trichoderma* green mould control in button mushroom farms. J. Agric. Sci..

[17] Abosriwil S.O., Clancy K.J. (2002). A protocol for evaluation of the role of disinfectants in limiting pathogens and weed moulds in commercial mushroom production. Pest Manag. Sci..

[18] Rinker D.L., Alm G., Van-Greuning M. (2008). Management of casing *Trichoderma* using fungicides. Science and Cultivation of Edible and Medicinal Fungi: Mushroom Science XVII, Proceeding of the 17th Congress of the International Society for Mushroom Science, Cape Town, South Africa, 20–24 May 2008.

[19] Romaine C.P., Royse D.J., Schlagnhaufer C. (2005). Superpathogenic *Trichoderma* resistant to Topsin M found in Pennsylvania and Delaware. Mushroom News.

[20] Bhardwaj G. (2003). Study of Growth Parameters of *Calocybe indica* (P & C). Ph.D. Thesis.

[21] Gupta A., Kerni P.N., Gupta A. (1995). In vitro evaluation of different chemicals against *T. viride* isolated from button mushroom. Res. Dev. Rep..

[22] Domondon D., Poppe J. (2000). Prevention of yield loss as influenced by *Trichoderma* in mushroom cultivation. Mededelingen van de Faculteit Landbouwkundige en Toegepaste Biologische Wetenschappen.

[23] Dhar B.L., Kapoor J.N. (1990). Inhibition of growth of *Agaricus bisporus* by systemic fungicides. Indian Phytopathol..

[24] Lovkesh B.J., Pahil V.S. (2006). Physiological and fungistatic interaction studies between *Pleurotus* spp. and *Trichoderma viride*. Crop Res. Hisar..

[25] Shandliya T.R., Guleria D.S. (1984). Control of green mould (*Trichoderma viride*) during the cultivation of *Agaricus bitorquis*. Indian J. Plant Pathol..

[26] Stanek M., Vojtechovska V. (1972). Test on the use of benomyl in mushroom cultivation. Mykol. Sb..

[27] Gandy D.G., Spencer D.M. (1974). Fungicide tolerance and its implications. Mushroom J..

[28] Geijin J., Van D. (1977). Practical control of *Verticillium* and *Mycogone*. Champignon.

[29] Kim G.P., Seok Y.S., Shin G.C., Paek Y.H. (1978). Studies on the control of *Mycogone perniciosa* Magn. In cultivated mushroom *(Agaricus bisporus* (Lange) Sigh.). Kor. J. Mycol..

