# Supplementary material for: Management of Green Mold Disease in White Button Mushroom (Agaricus bisporus) and Its Yield Improvement
Source: J Fungi (Basel). 2022 May 24;8(6):554. doi: 10.3390/jof8060554 (PMC9225557; doi:10.3390/jof8060554)
Supplement: Supplementary file 1 [file jof-08-00554-s001.zip › jof-1701106-supplementary.pdf]

Supplementary Materials: Table S1: Table represents mean sum of squares of non-systemic fungicides; Table S2: Table of mean sum of squares of systemic fungitoxicants

Table S1: Mean sum of squares of Non Systemic fungicides:

Source of Variation	Degree of Freedom	Mean Sum of square	Grand Mean	C.V.
**Effect of non-systemic fungicides on Intensity(%) of green mould disease of white button mushroom**				
**ENVIRONMENT-I**				
Treatment	10	2.491*	2.153	0.15
**ENVIRONMENT-II**				
Treatment	10	2.949*	2.305	0.20
**Pooled**				
Treatment	10	0.379*	2.229	0.21
Environment	1	5.399*		
Interaction	10	0.409*		
**Effect of Non Systemic fungicides on number of fruit bodies**				
Treatment	10	9.759*	92.73	0.04
**Effect of Non Systemic fungicides on fruit bodies weight**				
Treatment	10	2.606*	10.64	0.19
**Effect of Non Systemic fungicides on button yield**				
Treatment	10	20.86*	12.01	0.16
**Effect of Non Systemic fungicides on weight of pileus**				
Treatment	10	1.232*	6.687	0.30
**Effect of Non Systemic fungicides on diameter of pileus**				
Treatment	10	0.326*	3.584	0.56
**Effect of Non Systemic fungicides on weight of stipe**				
Treatment	10	0.107*	4.539	0.44
**Effect of Non Systemic fungicides on diameter of stipe**				
Treatment	10	0.492*	1.262	1.58
**Effect of Non systemic fungicides on *Trichoderma harzianum***				
Treatment	5	7.657*	48.85	3.6
**Effect of Non systemic fungicides on *Agaricus bisporus***				
Treatment	5	10813*	18.07	2.7

*** Significance at 5%**

Table S2: Mean sum of squares of Systemic Fungitoxicants:

Source of Variation	Degree of Freedom	Mean Sum of square	Grand Mean	C.V.
**Effect of systemic fungicides on Intensity (%) of green mould disease of white button mushroom**				
**ENVIRONMENT-I**				
Treatment	10	2.180*	2.726	0.12
**ENVIRONMENT-II**				
Treatment	10	2.431*	2.462	0.16
**Pooled**				
Treatment	10	4.568*	2.594	0.14
Environment	1	1.151*		
Interaction	10	0.442*		
**Effect of Systemic fungicides on number of fruit bodies**				
Treatment	10	29.65*	93.88	0.04
**Effect of Systemic fungicides on fruit bodies weight**				
Treatment	10	0.315*	10.74	0.19
**Effect of Systemic fungicides on button yield**				
Treatment	10	11.45*	10.48	0.16
**Effect of Systemic fungicides on weight of pileus**				
Treatment	10	0.354*	6.039	0.33
**Effect of Systemic fungicides on diameter of pileus**				
Treatment	10	0.137*	3.652	0.55
**Effect of Systemic fungicides on weight of stipe**				
Treatment	10	0.802*	4.768	0.42
**Effect of Systemic fungicides on diameter of stipe**				
Treatment	10	0.856*	1.311	1.53
**Effect of Systemic fungicides on *Trichoderma harzianum***				
Treatment	4	9.262*	58.72	1.75
**Effect of Systemic fungicides on *Agaricus bisporus***				
Treatment	4	6327*	21.18	1.3

*** Significant at 5%**
